# Strigolactones and Cytokinin Interaction in Buds in the Control of Rice Tillering

**DOI:** 10.3389/fpls.2022.837136

**Published:** 2022-07-01

**Authors:** Manrong Zha, Yanhui Zhao, Yan Wang, Bingxian Chen, Zecheng Tan

**Affiliations:** ^1^College of Biology Resources and Environmental Sciences, Jishou University, Jishou, China; ^2^Key Laboratory of Plant Resources Conservation and Utilization, College of Hunan Province, Jishou, China; ^3^Guangdong Key Lab for Crop Germplasm Resources Preservation and Utilization, Guangzhou, China

**Keywords:** rice, tiller bud, strigolactones, cytokinin, *OsCKXs*

## Abstract

Shoot branching is among the most crucial morphological traits in rice (*Oryza sativa* L.) and is physiologically modulated by auxins, cytokinins (CKs), and strigolactones (SLs) cumulatively in rice. A number of studies focused on the interplay of these three hormones in regulating rice tiller extension. The present study primarily aimed at determining the impact of different treatments, which were used to regulate rice tiller and axillary bud development on node 2 at the tillering stage and full heading stage, respectively. Transcription levels of several genes were quantified through qRT-PCR analysis, and an endogenous auxin and four types of CKs were determined through LC-MS/MS. Both nutrient deficiency and exogenous SL supply were found to inhibit rice tiller outgrowth by reducing the CK content in the tiller buds. Furthermore, supplying the inhibitor of both exogenous SLs and endogenous SL synthesis could also affect the expression level of *OsCKX* genes but not the *OsIPT* genes. Comparison of *OsCKX* gene expression pattern under exogenous SL and CK supply suggested that the induction of *OsCKX* expression was most likely via a CK-induced independent pathway. These results combined with the expression of CK type-A *RR* genes in bud support a role for SLs in regulating bud outgrowth through the regulation of local CK levels. SL functioned antagonistically with CK in regulating the outgrowth of buds on node 2, by promoting the *OsCKX* gene expression in buds.

## Key Messages

Strigolactones and cytokinins play antagonistically in the control of shoot branching in rice. The transcription level of *OsCKXs* was highly induced by SLs, suggesting an insight into the role of SLs in inhibiting the development of axillary buds.

## Introduction

Shoot architecture is a crucial morphological feature for plant survival and competition. It is among the key agronomic and major contributing factors to the yield and overall performance of rice (*Oryza Sativa* L.). As a major determinant of plant architecture, shoot branching involves the formation of axillary buds in the axils of leaves and subsequent outgrowth of buds. It is a well-known fact that shoot branching is strikingly affected by various environmental factors like drought and soil nutrient deficiencies (Horvath et al., 2003; Umehara et al., 2010; Sun et al., 2014; Luo et al., 2018). The initiation of axillary branching and development is a complex phenomenon and is found to be implicated with plant hormones like auxins, CKs, and SLs. Auxins and SLs are involved in inhibiting the bud outgrowth, while CK promotes it (Dun et al., 2009; Leyser, 2009; Beveridge and Kyozuka, 2010).

Rice tillering is strongly affected by nutrient availability, such as nitrogen (N) and phosphorus (P). N and P deficiency has been evidenced to increase SL content in roots and root exudates (Yoneyama et al., 2007; Xie et al., 2010; Brewer et al., 2013; Sun et al., 2014). The suppression of tiller bud outgrowth under P deficiency in wild-type plants will not happen in SL-deficient and signal mutants (Umehara et al., 2010). Based on previous studies, SLs were proved to be involved in the inhibition of rice tillering in response to N and P deficiency (Umehara et al., 2010; Xu et al., 2015). N deficiency inhibits the cell division-determined elongation (Luo et al., 2017), thus affecting the development of rice tiller buds. Meanwhile, N-controlled branching was proved partially by SLs, by using SL biosynthesis mutant in Arabidopsis (de Jong et al., 2014).

Strigolactones are a class of carotenoid-derived hormones that have been found in the root exudates of most plant species (Cook et al., 1966; Akiyama et al., 2005). Studies regarding SL biosynthesis and signaling mutants have indicated that SLs perform various roles in regulating plant development (Zou et al., 2006; Arite et al., 2007, 2009). The most featured role of SLs is in the regulation of axillary shoot branching in many plant species. Grafting studies demonstrated the synthesis of branch-inhibiting signals in the root or shoot tissues, and their subsequent movement in the upward direction (Domagalska and Leyser, 2011). This could result in inhibiting the bud outgrowth through branch-inhibiting signals or translocation of its precursors over long distances and may act locally in or near the axillary buds (Beveridge et al., 1996, 1997; Napoli, 1996; Morris et al., 2001; Turnbull et al., 2002; Booker et al., 2005; Simons et al., 2007). It is well-documented that the SMXL/D53 acts as a target for SL-induced D14-SCF-dependent protein degradation (Zhou et al., 2013; Jiang et al., 2013; Soundappan et al., 2015; Wang et al., 2015). In a broader sense, the second-messenger and canalization can be considered as two main models to study shoot branching regulation via SLs. As per the second-messenger model, root synthesized SLs and CKs are transported to shoot and directly affect branching in buds (Dun et al., 2012; Brewer et al., 2015). Auxin from apices acts indirectly to inhibit the release of dormant buds by regulating the synthesis of SLs and CKs in nearby tissues (Domagalska and Leyser, 2011). Both SLs and CKs affect branching by regulating *BRANCHED1* (*BRC1*, a major regulatory nexus for shoot branching) expression in buds. The *BRC1* expression is significantly reduced in the *max2* mutant and remarkably enhanced in *smxl6 smxl7 smxl8 max2* multiple mutants (Seale et al., 2017). In addition, exogenous SLs *rac*-GR24 supply can upregulate the *BRC1* expression rapidly, independent of any new protein synthesis, suggesting that *BRC1* is the most direct target of SL signaling (Dun et al., 2012).

Cytokinin is the only hormone that has been shown to promote axillary shoot branching. CK levels in the axillary bud can be affected by local auxin contents by regulating the expression of *adenosine phosphate-isopentenyl transferase* (*IPT*) genes (Turnbull et al., 1997; Shimizu-Sato et al., 2009). In addition, likely a trigger for bud release, CK was reported to promote auxin production and basipetal auxin transport out of the growing buds, consequently repressing the production of CK in the stem and limiting its availability for other buds (Bangerth et al., 2000; Tanaka et al., 2006; Shimizu-Sato et al., 2009).

Regarding the contrary roles of CKs and SLs in axillary bud outgrowth (Braun et al., 2012; Dun et al., 2012), the mechanism of their antagonistic functions on branching regulation is seldom studied. In pea and Arabidopsis, the CKs in the xylem sap of SL-deficient mutants were lower relative to wild-type plants due to feedback regulation operating in the SL branching pathways (Beveridge et al., 1997; Morris et al., 2001; Foo et al., 2007). The promotion of CK on axillary bud outgrowth can be reduced by exogenous SLs without affecting the CK biosynthesis genes in pea (Dun et al., 2012). Also, SL biosynthesis might be affected by CK, and the auxin induced the upregulation of *More Axillary Growth 4* (*MAX4*, SL biosynthesis gene), which can be prevented by CKs (Bainbridge et al., 2005). CKs and SLs may converge at a common point in the bud outgrowth regulation pathway in pea (Dun et al., 2012). In rice, CK levels in nodal tissues were found to be higher in D10-RNAi plants (presumed SL deficient) when compared to the wild-type plants (Zhang et al., 2010). Transcriptome analysis revealed that four *CKX* genes of Arabidopsis were downregulated in the *max2* mutant (SL mutant) (Ha et al., 2014). Recently, one of the *CKX* genes (*CKX9*) was promoted by SL signaling, thus leading to CK degradation in rice plants (Duan et al., 2019). On the other hand, CKs suppress biosynthesis in roots (Yoneyama et al., 2020), thus SLs and CKs could supress each other systemically.

In the current study, two different bud types of rice, tiller bud (located at fifth leaf axils) and node bud (located at the second nodes from the top), were investigated. Different treatments were applied to shift buds between the dormant stage and the transition stage. Auxin and CK content in both nodes and buds were measured using HPLC (LC-MS/MS). Also, hormone-related gene expression patterns, in response to each treatment, were also examined in this study. Our results gave an insight into how SLs integrate with CK to regulate bud outgrowth in rice tiller buds.

## Materials and Methods

### Plant Growth

The experiment was divided into two sessions according to different stages of treatment. Both sessions were conducted in the net house of Nanjing Agriculture University (Jiangsu Province, China) during the rice-growing season. An indicator cultivar, Yangdao 6, was used as a test crop in this study.

In the first group, 20-day-old rice seedlings (planted in a seedbed) were transplanted to 20 L plastic pots containing full nutrient solution with composition as described by Yoshida (1975). The nutrient solution contained 40 mg⋅L^–1^ CaCl_2_, 40 mg⋅L^–1^ K_2_SO_4_, 40 mg⋅L^–1^ MgSO_4_⋅7H_2_O, 0.5 mg⋅L^–1^ MnCl_2_⋅4H_2_O, 10 mg⋅L^–1^, NaH_2_PO_4_⋅2H_2_O, 0.05 mg⋅L^–1^ (NH_4_)_6_⋅Mo_7_O_24_⋅2H_2_O, 0.01 mg⋅L^–1^ ZnSO_4_⋅7H_2_O, 0.2 mg⋅L^–1^ H_3_BO_3_, 2.0 mg⋅L^–1^ FeCl_2_⋅6H_2_O, and 0.01 mg⋅L^–1^ CuSO_4_⋅5H_2_O. The N and P concentrations varied in the treatments depending upon the amounts of NH_4_NO_3_ and NaH_2_PO_4_⋅2H_2_O. The nutrient solutions of all treatments were treated with 0.1 M HCl to maintain a PH value of 5.5. In the second group, the rice seedlings of the same age were transplanted into plastic pots having diameter and height of 30 cm. Each pot, where four seedlings were transplanted, contained 15 kg of sieved soil. N (1.6 g plot^–1^ as urea), P (0.8 g plot^–1^ as single superphosphate), and potassium (1.2 g plot^–1^ as KCl) were applied at the time of seedling transplantation. Surface water was applied to irrigate these pots over the entire growing season of the plants.

### Plant Materials and Treatment

#### Tillering Stage

The rice plants were grown in a sufficient N (2.5 mM) and P (300 μM) concentration for sustainable growth. When these rice seedlings developed up to seven leaves on their main stems, they were divided into four treatment groups. One was treated with 2.5 mM N and 300 μM P (normal nutrient levels, Co-1 treatment), the second with 0.02 mM N and 300 μM P (LN treatment), the third with 2.5 mM N and 2 μM P (LP treatment), and the fourth with 2.5 mM N, 300 μM P, and a final concentration of 2 μM rac-GR24 (synthetic strigolactone, GR treatment).

As a reverse, some rice seedlings were grown under P-deficient conditions (2 μM). When these rice seedlings developed seven leaves on their main stems, they were divided into three treatment groups. One was treated with 2 μM P (Low P levels, Co-2 treatment), the second with 300 μM P (HP treatment), and the third with 2 μM P and a final concentration of 2 μM TIS108 (TIS treatment, as a potent and specific SL biosynthesis inhibitor).

#### Full Heading Stage

Different concentrations of BA (30 μM) and rac-GR24 (2 μM) were supplied in a volume of 0.5 ml directly to the axillary bud on node 2 as described by Gomez-Roldan et al. (2008) and Dun et al. (2009). Different from other plants, the bud on node 2 in rice is always covered by leaf sheaths. We peeled out the sheaths from the stem to pour the solution into the gap between the stem and sheaths. The sheath of control treatment (Co-3) was also peeled out as other hormone treatments but treated with solvent.

### Measurement of Endogenous Plant Hormones

The measurement of SL in root exudates was performed as described previously (Ishikawa et al., 2005; Umehara et al., 2008). For each sample, 50 ml of hydroponic culture medium loaded into a pre-treated Oasis HLB 3cc cartridge (Waters) was used after adding internal standard (1 ng of d_6_-5DS) and washing with de-ionized water. The SLs were determined using a UPLC-MS/MS analysis as described before (Zhou et al., 2013).

The extraction and purification of indole-3-acetic acid (IAA) and four types of CKs (tZ, tZR, iP, and iPR) were carried out using methods described by Dobrev and Kaminek (2002). The determination of IAA, tZ, tZR, iP, and iPR was performed using an LC-MS/MS (Aglient1290 and SCIEX-6500trap) system as previously described by Nakagawa et al. (2005).

### Gene Expression Analysis

Bud and node tissues were frozen, and total RNA isolation and cDNA synthesis were carried out as described in our previous work (Xu et al., 2015). Quantitative real-time PCR (qRT-PCR) was performed using the Roche LC 480 (Roche diagnostics, Penzberg, Germany) and SYBR *premix Ex Taq* Kit (TaKaRa). Each reaction contained 10 μL of SYBR *Premix Ex Taq*, 1 μL each of 10 μM gene-specific primer pair, 5 μL of template cDNA, and 4 μL of water. The thermal cycle of qRT-PCR was carried out at 95°C for 3 min, 40 cycles at 95°C for 15 s, and at 60°C for 60 s. Primer sequences used to amplify the transcripts are shown in Supplementary Table 1.

### Statistical Analysis

ANOVA test was performed using SPSS 17.0. Data from each sampling event were analyzed separately. Mean values were tested with the least significant difference test, and the significance level was set at *p* ≤ 0.05.

## Results

### Tiller Bud Growth Was Suppressed by LN, LP, and GR Treatment at Tillering Stage

**FIGURE 1:**
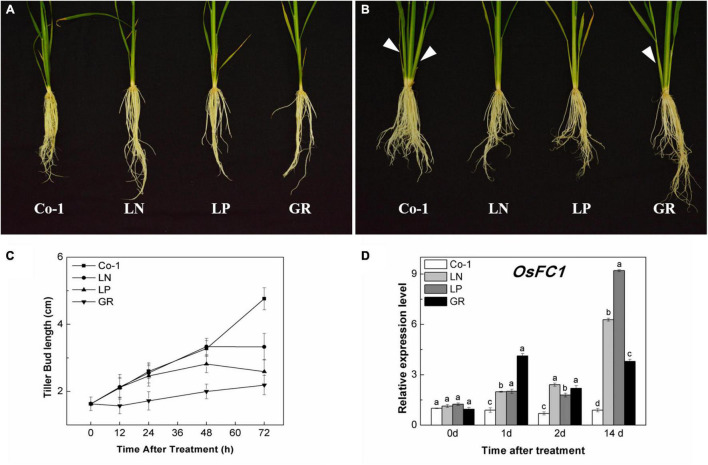
Effects of LN, LP, and GR treatments on the growth of rice tiller and OsFC1 expression in tiller bud. **(A)** Rice plant before treatment; **(B)** rice plants after 2 weeks of treatment; **(C)** outgrowth of rice tiller buds at the fifth leaf axils; and **(D)** OsFC1 expression in tiller buds at the fifth leaf axils. The arrows in **(A)** and **(B)** refer to the growth of new tillers within 2 weeks. Co-1 contains 2.5 mM N and 300 μM P in a nutrient solution, LN contains 0.02 mM N and 300 μM P in a nutrient solution, LP contains 2.5 mM N and 2 μM P in a nutrient solution, GR contains 2.5 mM N, 300 μM P, and 2 μM rac-GR24 in a nutrient solution. The expression of *OsFC1* in the tiller buds at 0, 1, 2, and 14 days after each treatment is represented relative to the Co-1 at 0 day. Vertical bars **(C)** represent mean ± standard error (*n* = 60). The value **(D)** obtained from the control treatment at 0 h after treatment was arbitrarily set at 1.0. Quantitative real-time PCR was performed in triplicate (three biological replicates), and mean values with SD are shown.

The tillers of the Co-1 plants grew naturally during the first 2 weeks of treatments; meanwhile, tillers were significantly reduced in response to nitrate deficiency (LN), phosphorous deficiency (LP), and exogenous rac-GR24 (GR) treatments (Figures 1A,B). The color of older leaves turned yellow in LN and LP plants, but remained green in GR plants, as in the case of Co-1 (Figures 1A,B). During 72 h of post-treatment, the growth of tiller buds of Co-1 remained active and grew normally. However, the tiller bud length in LN and LP plants was inhibited and found to be significantly shorter than those in the Co-1 plants at just 48 and 72 h of treatment, respectively (Figure 1C). Not surprisingly, the tiller buds in GR plants were inhibited significantly and showed growth stagnancy 12 h after treatment (Figure 1C). *OsFC1* is known to be a negative regulator of bud growth and works downstream of the SL signaling pathway. Consistent with the outcome, the transcription level of *OsFC1* was highly induced in LN, LP, and GR plants when compared to the Co-1 plants (Figure 1D).

### Cytokinin Content in Tiller Buds Was Reduced During Bud Inhibition by Strigolactones

**FIGURE 2:**
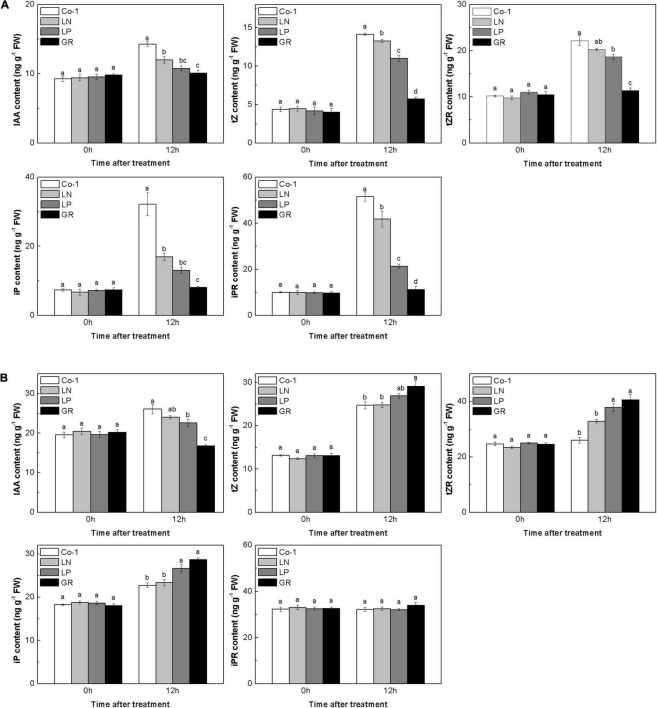
Effect of N, phosphorous, and GR24 on the amounts of CK and indole-3-acetic (IAA) in rice tiller buds **(A)** and nodes **(B)**. At 0 and 12 h after treatment, the amounts of CK and IAA in rice tiller buds located at the fifth leaf axils and nodes were measured (tZ, tZR, iP, iPR). FW, fresh weight. Values in each column at the same amount of hormone followed by different letters were significantly different at *p* = 0.05 (*n* = 3).

Auxins and CKs play a major role in regulating rice tiller outgrowth. To check whether the CK and auxin levels would change in rice tiller buds and nodes with nutrient deficiency and GR treatment, we measured the endogenous concentrations of several natural CKs and auxins in both tiller nodes and buds. In Co-1 control plants, the contents of IAA and CK in tiller buds and nodes changed after 12 h, which may be due to the plant growth during the daytime. The amounts of IAA decreased profoundly in GR and LP plants in both tiller buds and tiller nodes, and only decreased in tiller nodes in LN plants when compared to Co-1 plants (Figure 2). All forms of CKs, in reponse to GR treatment, showed decreased concentrations than those observed in Co-1 treatment after 12 h of treatment in tiller buds, indicating that exogenous SLs can reduce the amount of CKs in tiller buds (Figure 2A). In response to LN and LP treatments, the CK levels were observed to be lower than in Co-1 plants but not as significant as in GR plants in tiller buds. However, the CK concentration in tiller nodes did not show similar trends as noticed in tiller buds (Figure 2B). The tZ, tZR, and iPR contents were induced by GR, but only tZR and iP produced enhanced response to LP in tiller nodes. Interestingly, the iPR treatment was not affected by all the treatments and remained at a stable level with Co-1 in tiller nodes (Figure 2B).

**FIGURE 3:**
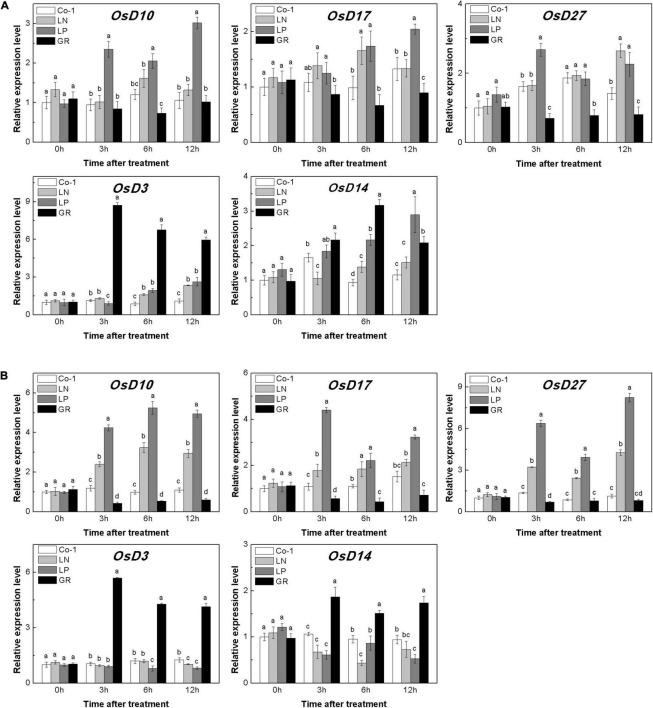
Expression patterns of *OsD10*, *OsD17*, *OsD27*, *OsD3*, and *OsD14* ST biosynthesis and signaling genes in rice tiller buds **(A)** located at the fifth leaf axils and root **(B)** expressed in response to the treatment. Total RNA was isolated from less than 0.1 g of buds and nodes each time. β-Actin was used as a reference gene. The value obtained from the control treatment at 0 h after treatment was arbitrarily set at 1.0. Expression of each gene at 0, 3, and 6 h after treatments is represented relative to the Co-1 treatment at 0 h. Quantitative real-time PCR was performed in triplicate (three biological replicates), and mean values with SD are shown.

The deficiency of both N and P led to an increase in the endogenous SL contents in rice roots (Sun et al., 2014). In our study, LN and LP significantly increased the expression of genes involved in the synthesis of SL in rice roots and tiller buds (Figure 3). The relative expression levels of *OsD10*, *OsD17*, and *OsD27* in both tiller nodes and buds were found to be higher in both LN and LP plants when compared to Co-1 plants, although the enhancement in the case of LP was more prominent than in LN (Figure 3). Interestingly, the expression levels of *OsD3* and *OsD14* were enhanced in tiller buds under GR compared with Co-1, but decreased in roots. Without a doubt, GR was found to be involved in the expression of SL signaling genes and negatively affected the transcription of SL synthesis genes significantly (Figure 3). All these results were consistent with the previous study on SL-related gene response to nutrient deficiency and exogenous SL treatment (Sun et al., 2014). Further, measurement of 2′-epi-5-deoxystrigol (epi-5DS), a native SL of rice, in the root exudates proved that none of the P treatments significantly promoted the endogenous SL biosynthesis, but was inhibited by GR24 supply (Supplementary Figure 1). Combining all the above-mentioned findings, we hypothesized that the decreased content of CK in tiller buds under LN, LP, and GR treatments may be correlated with endogenous and exogenous SLs. Furthermore, SLs may inhibit tiller bud outgrowth partly by reducing the local CK content in the bud.

### Strigolactone Promotes Cytokinin Degradation in Rice Tiller Buds at Tillering Stage

**FIGURE 4:**
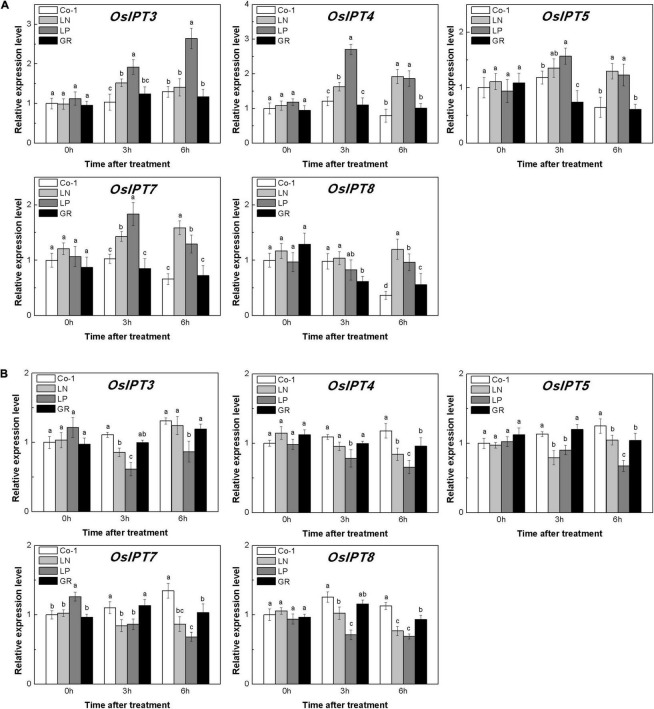
Expression patterns of *OsIPT3*, *OsIPT4*, *OsIPT5*, *OsIPT7*, and *OsIPT8* CK biosynthesis genes in rice tiller buds **(A)** and tiller nodes **(B)** located at the fifth leaf axils expressed in response to the treatment. Total RNA was isolated from less than 0.1 g of buds and nodes each time. β-Actin was used as a reference gene. The value obtained from the control treatment at 0 h after treatment was arbitrarily set at 1.0. Quantitative real-time PCR was performed in triplicate (three biological replicates), and mean values with SD are shown.

**FIGURE 5:**
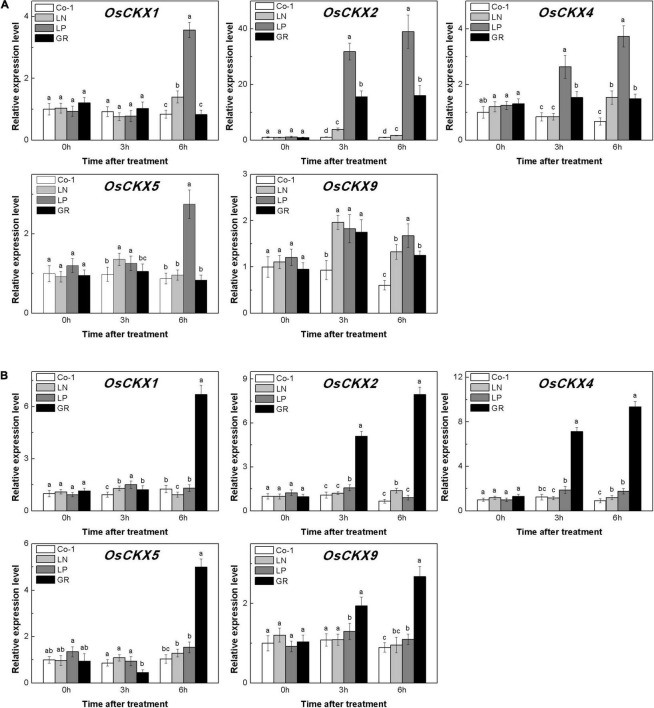
Expression patterns of *OsCKX1*, *OsCKX2*, *OsCKX4*, *OsCKX5*, and *OsCKX9* CK degradation genes in rice tiller buds **(A)** and tiller nodes **(B)** located at the fifth leaf axils expressed in response to the treatment. Total RNA was isolated from less than 0.1 g of buds and nodes each time. β-Actin was used as a reference gene. The value obtained from the control treatment at 0 h after treatment was arbitrarily set at 1.0. Quantitative real-time PCR was performed in triplicate (three biological replicates), and mean values with SD are shown.

The level of CKs is controlled by both CK biosynthesis and degradation in rice. Adenosine phosphate-isopentenyl transferase (IPT) catalyzes the rate-limiting step of CK biosynthesis. In Arabidopsis, *AtIPTs* respond specifically to NO^3–^ and NH^4+^ treatments (Takei et al., 2004; Sakamoto et al., 2006). Auxin moves basipetally and controls local CK biosynthesis by mediating *PsIPT* expression in pea plants (Li et al., 1995; Tanaka et al., 2006). Eight *OsIPT*s genes were revealed by molecular and biochemical studies in the rice genome (Sakamoto et al., 2006). The *CKX* encoding genes control the level of endogenous CKs, which are required for irreversible CK degradation in plants and play an indicating role in detecting CK levels (Galuszka et al., 2001; Ha et al., 2012). The CKX enzymes, encoded by the multigene family, include 11 *OsCKXs* in rice. We only successfully detected five *OsIPTs* and five *OsCKXs* in our samples. The expression of the most detected *OsIPT* genes was increased by more than 1.5 times in rice tiller buds but decreased quickly in tiller nodes in response to LN and LP (Figure 4). However, GR did not change significantly the expression level of five *OsIPT* genes in either tiller nodes or buds (Figure 4). All the five detected *OsCKX* genes were significantly upregulated by LP, but only *OsCKX2, OsCKX4*, and *OsCKX9* increased by more than two times in the rice tiller buds of LN plants (Figure 5A). In tiller nodes, LN and LP did not significantly affect the OsCKX gene expression (Figure 5B). Three *OsCKX* genes, except for *OsCKX1* and *OsCKX5*, were enhanced by GR, while only GR prominently induced the expression of five *OsCKX* genes in tiller nodes (Figure 5B). These results suggest that SLs can decrease the CK content in buds by increasing CK degradation, whereas only exogenous SLs functioned in tiller nodes.

**FIGURE 6:**
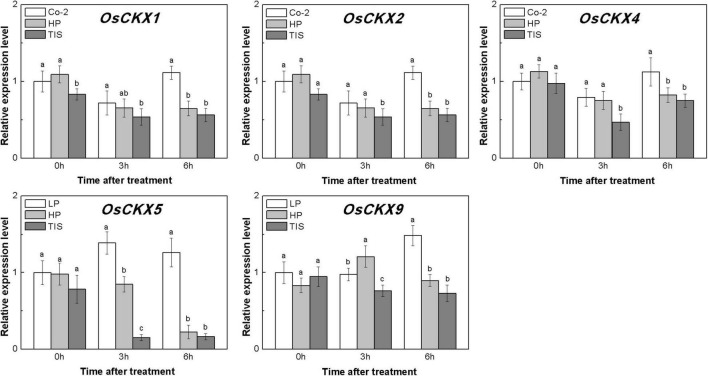
Expression patterns of *OsCKX1*, *OsCKX2*, *OsCKX4*, *OsCKX5*, and *OsCKX9* CK degradation genes in rice tiller buds located at the fifth leaf axils expressed in response to the treatment. Co-2 contains 2 μM P in a nutrient solution, HP contains 300 μM P in a nutrient solution, and TIS contains 2 μM P and 2 μM TIS108 in a nutrient solution. Total RNA was isolated from less than 0.1 g of buds and nodes each time. β-Actin was used as a reference gene. The value obtained from the control treatment at 0 h after treatment was arbitrarily set at 1.0. Quantitative real-time PCR was performed in triplicate (three biological replicates), and mean values with SD are shown.

To confirm our hypothesis, reverse experiments were designed using high phosphorous (HP) treatment and TIS108 (a neogenesis SL synthesis inhibitor) supply. The TIS108 is a specific SL biosynthesis inhibitor that inhibits SL biosynthesis in both rice and Arabidopsis (Ito et al., 2011, Ito et al., 2013), but its target site is still unknown. HP or TIS108 supply could release the tiller buds from dormancy and reduce endogenous SLs biosynthesis in rice, based on low P growth conditions (Supplementary Figures 2, 3). With these treatments, five *OsCKXs* genes were found to be reduced in different degrees (Figure 6). At the same time, the transcription levels of *OsIPT3*, *OsIPT4*, and *OsIPT7* in tiller buds increased more than 1.5 times in response to HP when compared to Co-2 plants (Supplementary Figure 4). However, the transcription level of five *OsIPT* genes remained at a relatively stable level in response to TIS in tiller buds (Supplementary Figure 4). Under the conditions of nutrient deficiency and GR, it emerged that SLs functioned as regulators of CK degradation in rice tiller bud. In a previous study, exogenous SL supply appeared to be reducing bud growth in response to CK in pea (Dun et al., 2012). Furthermore, impaired SL signaling led to the downregulation of *CKX*-encoding genes (Ha et al., 2014). All these results can support our notion that SL promotes CK degradation in rice buds.

### Exogenous Strigolactone Supply Reduces the Cytokinin Promotion of Bud Growth Directly at Full Heading Stage

**FIGURE 7:**
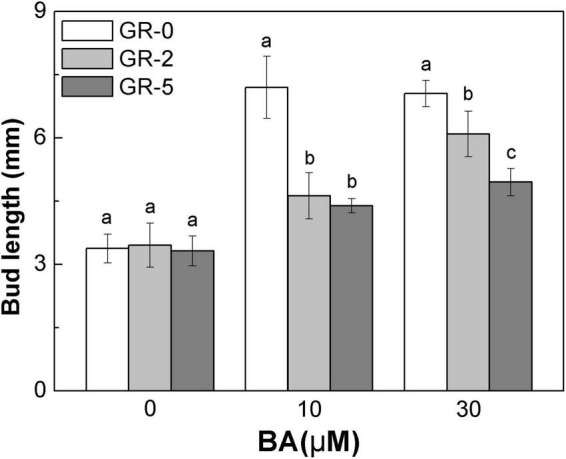
Strigolactone reduces the stimulatory effect of BA-induced bud outgrowth in rice. rac-GR24 and/or BA were supplied to the axillary bud directly on node 2 at the full heading stage. Bud length of 60 buds at node 2 was measured 3 days after treatment. Vertical bars in C represent mean ± standard error values (*n* = 60).

To further explore the relationship between SLs and CK degradation in rice bud inhibition, we shifted our attention to rice node 2 at the full heading stage, to enable focusing on each specific bud. At full heading stage, all the buds at node 2 no longer outgrew and maintained a dormant state unless stimulated by the environmental conditions, such as decapitated and plant hormone supply. Buds on node 2 were released from the dormant state and grew out with BA supply (Figure 7). A direct supply of GR24 to the bud resulted in a reduction of BA-induced bud growth in rice, and the reduction was enhanced with the increase amonts of GR24 (Figure 7). This finding demonstrated that SLs and CK played antagonistic roles in the regulation of bud outgrowth in rice.

**FIGURE 8:**
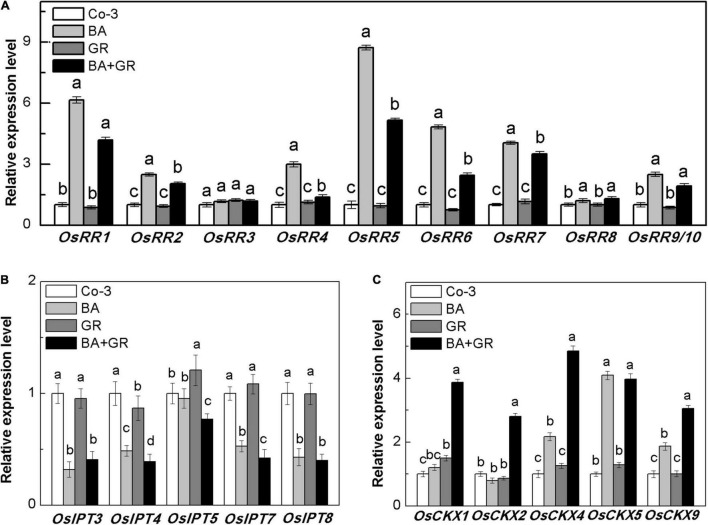
Effect of GR or/and BA treatments on CK-related gene expression in axillary buds at node 2 at full heading stage. **(A)** Cytokinin-type-A RR genes expression, **(B)** five isopentenyl transferase genes expression, and **(C)** five cytokinin oxidase/dehydrogenase genes expression. The axillary bud at node 2 was treated for 12 h with or without BA (40 μM) or/and rac-GR (2 μM). The expression of each gene in the bud at node 2 is represented relative to Co-3. Total RNA was isolated from less than 0.1 g of buds and nodes each time. β-Actin was used as a reference gene. The value obtained from the control treatment at 0 h after treatment was arbitrarily set at 1.0. Quantitative real-time PCR was performed in triplicate (three biological replicates), and mean values with SD are shown.

The outgrowth of axillary bud has been well-confirmed and is correlated with the local CK concentration in rice, where the CK acted independently to regulate the bud growth (Chatfield et al., 2000). The genes *type-A RRs* have been defined as the CK-mediated genes that are required for bud activation in Arabidopsis (Müller et al., 2015). Also, such genes were used as marker genes of CK-inducible processes (López-Bucio et al., 2007). To determine whether the exogenous SLs reduced the bud outgrowth via the CK pathway directly, the transcript levels of ten *OsRR* genes were detected with the interaction of BA and GR24. Most of the *OsRR* genes increased on treatment with BA and reduced up to different extents following the application of GR24 (Figure 8A), suggesting that the exogenous SL supply may act upon the CK-mediated bud activation pathway.

To determine if the observed reduction of *OsRR* gene expression was directly due to CK biosynthesis or degradation, the expression of *OsIPT* and *OsCKX* genes was also observed in the bud on node 2 (Figures 8B,C). There was a significant reduction in the expression of *OsIPT* genes, which may be due to the feedback regulation of exogenous BA supply. With or without BA, the exogenous SLs and GR24 did not affect the expression of *OsIPT* genes. As expected, the transcription level of *OsCKX* genes was highly induced by GR treatment in the presence of BA (Figure 8C). The endogenous CK contents in the bud on node 2 were profoundly induced by BA and reversed by GR combined treatment (Supplementary Figure 5). Our result, here, supported the hypothesis that SLs inhibit bud growth by inducing CK degradation in rice, and the effectiveness of SL treatment depends on the local content of CK.

## Discussion

### Nutrient Deficiency and rac-GR24 Supply Inhibited Rice Tiller at Tillering Stage

Our results for rice were consistent with substantial evidence that N and P deficiencies inhibit axillary buds to grow out in many species (Figure 1). The long-term inhibition of GR on tillering was not as significant as observed in LN and LP treatments (Figures 1A,B), which may be due to the unstable characteristics of rac-GR24 in water (Akiyama et al., 2010; Bromhead et al., 2015). The tiller bud growth had a quicker response to GR than to LN and LP (Figure 1C). The inhibited tiller phenotype under LN, LP, and GR conditions might be related to the *OsFC1* increase in tiller buds (Figure 1D). The expression level of *OsFC1* appeared to be under hormonal control, thus manipulating shoot branching (Minakuchi et al., 2010; Braun et al., 2012). Nutrient deficiencies elevated SL biosynthesis and exudation in roots, which is suggested to be one of the factors responsible for tiller suppression (Koltai et al., 2010; Kapulnik et al., 2011a,b; Ruyter-Spira et al., 2011). These reports were consistent with our finding that LN and LP can increase endogenous SL biosynthesis and signaling in both nodes and tiller buds. Together, exogenous SL supply and nutrient deficiency-induced endogenous SLs acted in the same manner in the inhibition of rice tiller bud growth.

### Cytokinin Contents Show a Decreasing Response to Both Exogenous and Endogenous Strigolactones in Tiller Buds

Strigolactone exudation was enhanced by low P and low N concentrations (Yoneyama et al., 2007, 2012; Jamil et al., 2011a,b; Sun et al., 2014). By using nutrient deficiency and GR24 treatment, we were able to create increased levels of endogenous SLs and exogenous SLs, respectively (Figure 3). Here, we showed that LN, LP, and GR induced a decrease in the contents of all the four endogenous CKs only in the rice tiller buds when compared to that observed in the control treatment (Figure 2). CK content in buds can be well correlated with the outgrowth of. A direct application of endogenous CK to buds causes the activation of axillary buds (Pillay and Railton, 1983; Cline et al., 1997). Above all, we hypothesized that the decreased levels of CK concentrations in tiller buds, in case of nutrient deficiencies and GR24 treatment, might be the reason for the inhibition of bud formation.

Plenty of evidence suggested that SLs may affect the levels of CK. CK levels in root xylem sap decreased in both SL-deficient and response mutants in pea and Arabidopsis (Foo et al., 2007; Waldie et al., 2010). In *D10*-RNAi rice plants, the CK level in nodal tissues was increased and led to a longer growth of local buds than observed in the wild type (Zhang et al., 2010). All these findings suggest that the effect of SLs on CK levels is diverse in different tissues. Also, decreased CK content induced by SLs in buds was not caused by the delivery of lower CK amounts from tiller nodes to buds, as reported by Dun et al. (2012). So, it is not surprising that even CK content shows converse results between tiller buds and nodes (Figure 2). Based on a previous study, endogenous IAA could decrease the biosynthesis of CK (Nordström et al., 2004; Shimizu-Sato et al., 2009). The reduced level of IAA may likely be the reason that led the CK contents to an increment in roots and then transport to the tiller node after 12 h of treatment.

### Endogenous Strigolactone and Exogenous rac-GR24 Promote the Cytokinin Degradation

The CK is recognized as an essential regulator of both the plant root system and shoot branching. Previous work suggested that exogenous GR24 promoted CK degradation in rice (Sun et al., 2014). Also, *OsCKX9* was proved to be activated by SLs signaling, and thus promotes CK degradation in rice. In this study, the expression of *OsIPT* genes showed different transcription patterns in tiller nodes and tiller buds under LN and LP conditions at tillering stage. But GR does not change the biosynthesis pattern in both rice tiller nodes and tiller buds. Consistent with our hypothesis, CK biosynthesis was not affected by GR24 supply in pea plants (Dun et al., 2012). Five *OsCKX* genes were significantly induced by GR24 supply and even by LP in our results. However, GR treatment and nutrient deficiency displayed different effects on the expression levels of *OsIPTs* and *OsCKXs* (Figures 4, 5), suggesting the different modes of action response to these treatments. This is probably because the exogenous SLs may function only through SL signal transduction, but nutrient deficiency is linked to multiple signals including SL signaling.

Furthermore, we examined the effect of reduced endogenous SLs on the expression of *OsCKX* genes. As expected, HP and TIS decreased the expression of *OsCKX* genes in the tiller bud. These reports indicated that both endogenous and exogenous SLs may, somehow, regulate the expression of *OsCKX* genes in rice. Above all, these five increased/reduced *OsCKX* genes corresponded to local CK levels in rice tiller buds.

### Strigolactones and Cytokinin Act Antagonistically on the Rice Buds Outgrowth

The supply of exogenous SLs and CK to the buds on node 2 at the full heading stage (Figure 7) showed that SLs and CK acted antagonistically on bud outgrowth. This was also confirmed in other species like pea and Arabidopsis (Braun et al., 2012; Dun et al., 2012), but never in rice. GR24 supply can suppress the increment of *OsRR* gene expression induced by BA. The *type-A RR* genes are the targets of CK signaling for primary transcription and rapid response (Brandstatter and Kieber, 1998; Imamura et al., 1998; D’Agostino et al., 2000; Jennifer et al., 2007). These findings suggested that the inhibition function of GR24 may be partly attributed to the impairment of CK downstream reaction.

We further explored the CK biosynthesis and degradation mechanisms with the BA and GR24 treatment (Figure 8). Consistent with the results we got at tillering stage, combined treatment of BA and GR24 induced an intermediate enhancement in the CK degradation, rather than exhibited by the CK biosynthesis change alone in buds on node 2. However, the application of GR24 alone to the buds did not change the expression of *OsCKX* genes in dormant buds, suggesting that local CK was required in the process of GR24 promoted expression of *OsCKX* genes.

Exogenous CK supply on buds directly induced the endogenous CK in buds and subsequently induced a negative feedback loop of CK biosynthesis, resulting in increased CK degradation. The combined GR24 supply showed an enhancement of CK degradation, but no changes were observed in CK biosynthesis. The expression pattern of *OsCKX* genes was partly different in response to BA and GR treatment. Together with the expression of the *OsRR* genes, we hypothesized that exogenous GR24 supply promoted the CK degradation partly by an independent pathway, and not just by promoting CK-induced CK degradation.

### Interaction Between Strigolactones, Cytokinin, and Auxins in the Control of Shoot Branching

Tiller outgrowth in rice is the combined function of a number of contributing factors, including varying levels of different plant hormones in the growing buds. SLs and CKs were implicated in the regulation of bud outgrowth. In this paper, we hypothesized that the SLs act directly in bud to inhibit the axillary bud outgrowth by promoting the local CK degradation. However, CK can also act independently of *PsBRC1*, as in pea, CK can still promote branching even in *psbrc1* mutants (Braun et al., 2012). The rice *OsFC1* transcription factor, which was implicated in bud outgrowth, was upregulated by rac-GR24 and downregulated by BA in rice axillary buds in our previous work (Xu et al., 2015). As described in the second-messenger model, SLs can regulate the expression level of *OsFC1* directly via the SL signaling pathway. Alternatively, SLs could also affect the *OsFC1* transcription level through CK signaling pathway by promoting the degradation of CK in the bud. Besides, CK can also act independently of *PsBRC1* (*OsFC1* in rice), as in pea, CK can still promote bud outgrowth even in *brc1* mutants (Braun et al., 2012). It was reported that the change in the local CK level in the bud always preceded the auxin efflux from the bud (Shimizu-Sato et al., 2008), which supports the notion that auxin transport is important for continued bud growth after bud release (Hayward et al., 2009; Prusinkiewicz et al., 2009; Crawford et al., 2010).

## Data Availability Statement

The original contributions presented in this study are included in the article/Supplementary Material, further inquiries can be directed to the corresponding author/s.

## Author Contributions

MZ designed the research and YW improved it. MZ performed research. MZ and ZT analyzed the data. MZ and YZ wrote the manuscript. YW and BC gave useful suggestions. All authors contributed to the article and approved the submitted version.

## Conflict of Interest

The authors declare that the research was conducted in the absence of any commercial or financial relationships that could be construed as a potential conflict of interest.

## Publisher’s Note

All claims expressed in this article are solely those of the authors and do not necessarily represent those of their affiliated organizations, or those of the publisher, the editors and the reviewers. Any product that may be evaluated in this article, or claim that may be made by its manufacturer, is not guaranteed or endorsed by the publisher.

## Abbreviations

SLs: strigolactones
BA 6: benzyladenine
CK: cytokinin
CKX: cytokinin oxidase/dehydrogenase
HPLC: high-performance liquid chromatography
iP: isopentenyladenine
iPR: isopentenyladenine riboside
tZ: *trans*-zeatin
tZR: *trans*-zeatin riboside
ARR: type-A Arabidopsis response regulators.
