# Supplementary material for: Strigolactones and Cytokinin Interaction in Buds in the Control of Rice Tillering
Source: Front Plant Sci. 2022 Jul 1;13:837136. doi: 10.3389/fpls.2022.837136 (PMC9286680; doi:10.3389/fpls.2022.837136)
Supplement: Supplementary file 1 [file Data_Sheet_1.PDF]

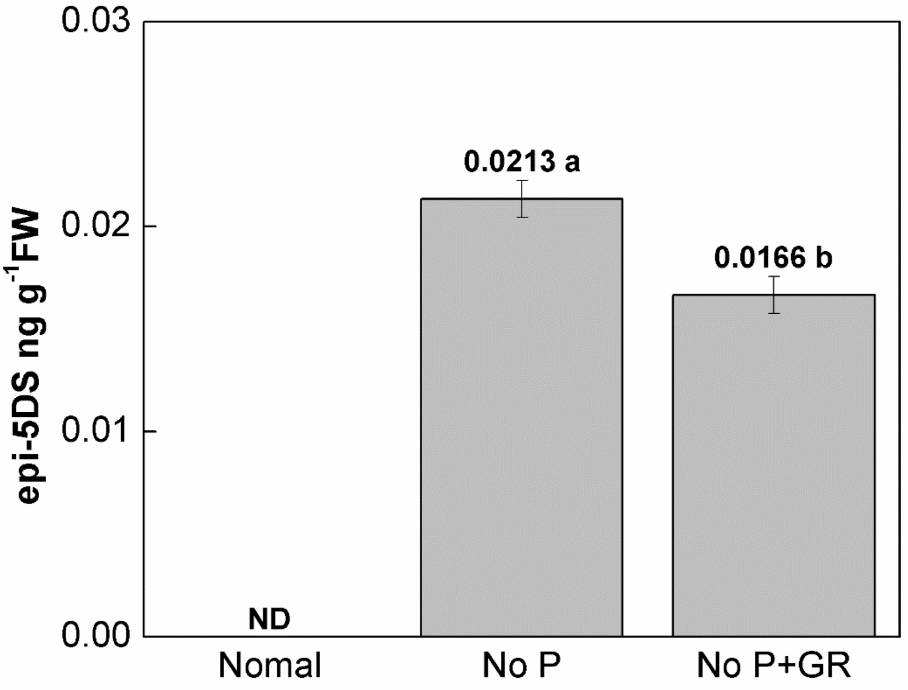

Supplementary Fig. S1 Liquid chromatography–tandem mass spectrometry (LC–MS/MS) measurement of epi-5DS levels in root exudates. Normal, regular nutrient solution; No P, nutrient solution without P; No P+GR, nutrient solution without P with 2uM GR24 gfw, per gram fresh weight; ND, not detected. Values are means ± s.d. (n=3).
