# Supplementary material for: Strigolactones and Cytokinin Interaction in Buds in the Control of Rice Tillering
Source: Front Plant Sci. 2022 Jul 1;13:837136. doi: 10.3389/fpls.2022.837136 (PMC9286680; doi:10.3389/fpls.2022.837136)
Supplement: Supplementary file 2 [file Data_Sheet_2.PDF]

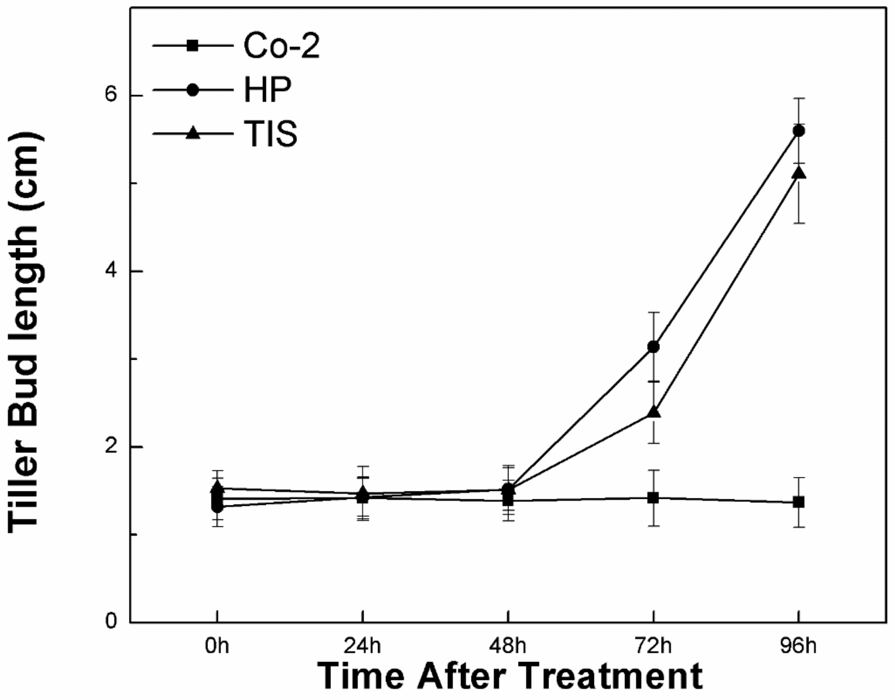


**Supplementary Fig. S2** Effects of HP and TIS treatment on the growth of rice tiller buds at the fifth leaf axils. Vertical bars represent mean ± standard error (n=40). Co-2, 2 μM P in nutrient solution, HP, 300 μM P in nutrient solution, TIS, 2 μM P and 2 μM TIS108 in nutrient solution.
