# Supplementary material for: Strigolactones and Cytokinin Interaction in Buds in the Control of Rice Tillering
Source: Front Plant Sci. 2022 Jul 1;13:837136. doi: 10.3389/fpls.2022.837136 (PMC9286680; doi:10.3389/fpls.2022.837136)
Supplement: Supplementary file 3 [file Data_Sheet_3.PDF]

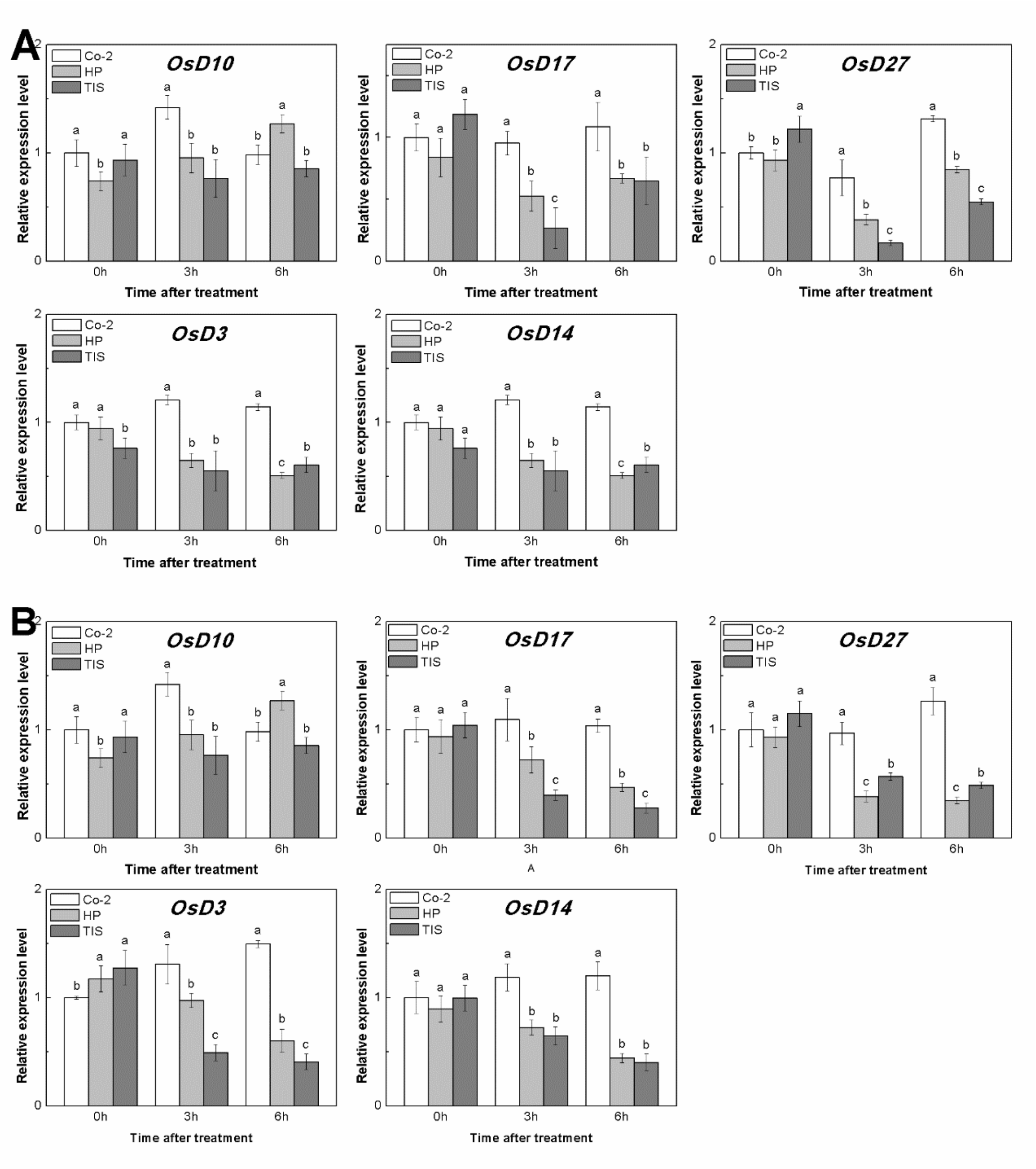

Supplementary Fig. S3 Expression patterns of *OsD10*, *OsD17*, *OsD27*, *OsD3* and *OsD14* strigolactone-biosynthesis and -signalling genes in rice tiller bud (A) located at the fifth leaf axils and root (B) expressed in response to the treatment. Total RNA was isolated from less than 0.1 g buds and nodes each time. β-Actin was used as a reference gene. The value obtained from the control treatment at 0 h after treatment was arbitrarily set at 1.0. Quantitative real-time PCR was performed in triplicate (three biological replicates) and mean values with SD are shown.
