# Supplementary material for: Strigolactones and Cytokinin Interaction in Buds in the Control of Rice Tillering
Source: Front Plant Sci. 2022 Jul 1;13:837136. doi: 10.3389/fpls.2022.837136 (PMC9286680; doi:10.3389/fpls.2022.837136)
Supplement: Supplementary file 4 [file Data_Sheet_4.PDF]

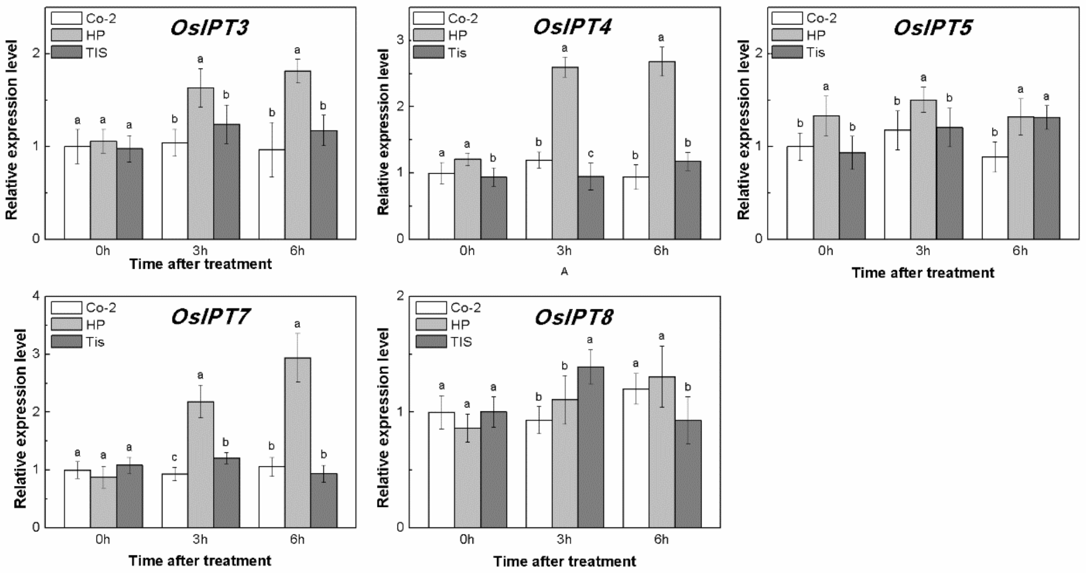

Supplementary Fig. S4 Expression patterns of *OsIPT3*, *OsIPT4*, *OsIPT5*, *OsIPT7* and *OsIPT8* cytokinin –biosynthesis genes in rice tiller bud located at the fifth leaf axils expressed in response to the treatment. Co-2 contain 2 μM P in nutrient solution, HP contain 300 μM P in nutrient solution, TIS contain 2 μM P and 2 μM TIS108 in nutrient solution. Total RNA was isolated from less than 0.1 g buds and nodes each time. β-Actin was used as a reference gene. The value obtained from the control treatment at 0 h after treatment was arbitrarily set at 1.0. Quantitative real-time PCR was performed in triplicate (three biological replicates) and mean values with SD are shown.
