# Supplementary material for: Strigolactones and Cytokinin Interaction in Buds in the Control of Rice Tillering
Source: Front Plant Sci. 2022 Jul 1;13:837136. doi: 10.3389/fpls.2022.837136 (PMC9286680; doi:10.3389/fpls.2022.837136)
Supplement: Supplementary file 5 [file Data_Sheet_5.PDF]

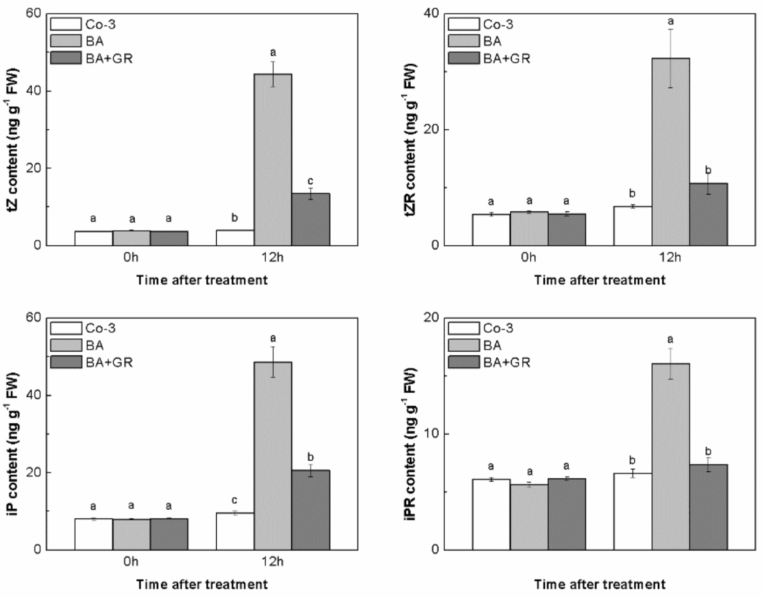

Supplementary Fig. S5 Effect of BA and GR24 on amounts of cytokinin(CK) in buds on node 2. At 0, 12 h after treatment, the amount of CK in rice buds located at the node 2 were measured tZ trans-zeatin, tZR trans-zeatin riboside, iP isopentenyl adenine, iPR iP riboside. FW fresh weight. Values in each column at the same amount of hormone followed by different letters were significantly different at p = 0.05 (n = 3)
